# 一步式QuEChERS-气相色谱-三重四极杆质谱法快速测定风干牦牛肉中15种*N*-亚硝胺

**DOI:** 10.3724/SP.J.1123.2023.12009

**Published:** 2024-05-08

**Authors:** Han XIA, Kaixuan TONG, Zhehui ZHU, Yujie XIE, Xingqiang WU, Qiaoying CHANG, Hongyi ZHANG, Chunlin FAN, Hui CHEN

**Affiliations:** 1.中国检验检疫科学研究院, 北京 100176; 1. Chinese Academy of Inspection and Quarantine, Beijing 100176, China; 2.河北大学化学与材料科学学院,河北 保定 071002; 2. College of Chemistry and Materials Science, Hebei University, Baoding 071002, China; 3.西藏自治区产品质量监督检验所, 西藏 拉萨 850000; 3. Tibet Product Quality Supervision and Inspection Institute, Lhasa 850000, China

**Keywords:** 一步式QuEChERS, 气相色谱-串联质谱, *N*-亚硝胺, 风干牦牛肉, one-step QuEChERS, gas chromatography-tandem mass spectrometry (GC-MS/MS), *N*-nitrosamines, air-dried yak meat

## Abstract

建立了一步式QuEChERS自动提取和净化技术结合气相色谱-串联质谱同时测定风干牦牛肉中15种*N*-亚硝胺的分析方法。样品水化后,经乙腈提取,加入4.0 g MgSO_4_和1.0 g NaCl除水,经十八烷基硅烷(C_18_)和*N*-丙基乙二胺(PSA)填料净化,采用DB-HeavyWAX色谱柱(30 m×0.25 mm×0.25 μm)分离15种*N*-亚硝胺,在多重反应监测(MRM)模式下进行测定,外标法定量。结果表明,15种*N*-亚硝胺分离性能良好,在0.1~200 μg/L范围内线性关系良好,相关系数(*r*^2^)≥0.9990;检出限(LOD)为0.05~0.20 μg/kg,定量限(LOQ)为0.10~0.50 μg/kg; 在1倍、2倍和10倍LOQ 3个添加水平下的平均回收率分别为79.4%~102.1%、80.6%~109.5%、83.0%~110.6%,相对标准偏差(RSD)为0.8%~16.0%。应用建立的方法检测2种不同加工工艺的市售样品,其中7种*N*-亚硝胺类化合物(*N*-亚硝基二甲胺、*N*-亚硝基二异丁胺、*N*-亚硝基二正丁胺、*N*-亚硝基甲基苯胺、*N*-亚硝基乙基苯胺、*N*-亚硝基吡咯烷、*N*-亚硝基二苯胺)均有不同程度检出,平均含量为0.08~20.18 μg/kg,且熟制风干牦牛肉中*N*-亚硝胺的检出率和平均含量均高于传统生制风干牦牛肉。该方法实现了前处理的自动化,相较于其他传统方法,操作简单,实验效率高,人为影响因素小,检测灵敏度高,适用于风干牦牛肉中15种*N*-亚硝胺的快速测定,为研究肉制品中*N*-亚硝胺的测定提供了方法支持。

## Abstract (en)

A method based on gas chromatography-triple quadrupole mass spectrometry (GC-MS/MS) coupled with one-step QuEChERS technique was developed for the simultaneous determination of 15 *N*-nitrosamines in air-dried yak meat. The hydration volume, extraction solvent, extracting salt, and cleaning material were optimized according to the characteristics of the *N*-nitrosamines and sample matrix. The optimized conditions were as follows: 10 mL of purified water for sample hydration, acetonitrile as the extraction solvent for the sample after hydration, 4.0 g of anhydrous MgSO_4_ and 1.0 g of NaCl as extracting salts, 500 mg of MgSO_4_+25 mg of C_18_+50 mg of PSA as cleaning materials. Favorable recoveries of the 15 *N*-nitrosamines were obtained when the extraction solution was incompletely dried. Thus, the final extract was dried to below 0.5 mL under a mild nitrogen stream and then redissolved to 0.5 mL with acetonitrile. After filtration, 200 μL of the sample was transferred to an autosampler vial for GC-MS/MS analysis. The 15 *N*-nitrosamines were determined using GC-MS/MS on a DB-HeavyWAX column (30 m×0.25 mm×0.25 μm) with an electron impact ion source in multiple-reaction monitoring (MRM) mode, and quantified using an external standard method.

Under the optimized experimental conditions, the results showed that the calibration curves exhibited good linearities for the 15 *N*-nitrosamines, with correlation coefficients (*r*^2^) greater than 0.9990. The limits of detection (LODs) and the limits of quantification (LOQs) ranged from 0.05 to 0.20 μg/kg and from 0.10 to 0.50 μg/kg, respectively. At spiked levels of 1LOQ, 2LOQ, and 10LOQ, the average recoveries were 79.4%-102.1%, 80.6%-109.5%, and 83.0%-110.6%, respectively, and the relative standard deviations were in the range of 0.8%-16.0%. The low matrix effects of the 15 *N*-nitrosamines indicated the high sensitivity of the proposed method. The method was applied to detect representative commercial air-dried yak meat samples obtained using different processing techniques. Seven *N*-nitrosamines, including *N*-nitrosodimethylamine, *N*-nitrosodiisobutylamine, *N*-nitrosodibutylamine, *N*-methyl-*N*-phenylnitrous amide, *N*-ethyl-*N*-nitrosoaniline, *N*-nitrosopyrrolidine, and *N*-nitrosodiphenylamine were detected in all samples. The average contents of the seven *N*-nitrosamines was 0.08-20.18 μg/kg. The detection rates and average contents of the *N*-nitrosamines in cooked air-dried yak meat samples were higher than those in traditional raw air-dried yak meat samples. Compared with the manual QuEChERS method, the one-step QuEChERS method developed integrated the extraction and clean-up procedures into one single run, and the detection efficiency was considerably improved. The developed method is simple, rapid, highly sensitive, and insusceptible to human errors. Thus, it is useful for the determination of *N*-nitrosamines in air-dried yak meat and can be extended to the qualitative and quantitative analysis of *N*-nitrosamines in other meat products. It also provides method support and a data reference for the general determination of *N*-nitrosamines, which is of great significance for food safety.

*N*-亚硝胺(*N*-nitrosamines, *N*-NAs)与黄曲霉素、苯并芘是世界公认的三大致癌物质。自1956年Magee等^[1]^发现*N*-亚硝基二甲胺(NDMA)能使大鼠罹患肝癌以来,*N*-亚硝胺日益成为人们重视的致癌物。近几年,越来越多的流行病学研究证明*N*-亚硝胺及其前体物质的摄入量同直肠癌、胃癌、食管癌等癌症的发病风险成正相关^[2]^。*N*-亚硝胺是胺类与亚硝酸、亚硝酸盐或氮氧化物等反应而形成,广泛存在于各种肉制品^[3⇓-5]^、水产品^[6]^、水^[7,8]^和化妆品^[9,10]^中。目前已发现的*N*-亚硝胺类化合物有300多种,其中90%具有较强的致癌性^[11]^, NDMA和*N*-亚硝基二乙胺(NDEA)毒性最强,国际癌症研究机构(IARC)将其列为2A类致癌物,*N*-亚硝基甲乙胺(NMEA)、*N*-亚硝基二正丁胺(NDBA)、*N*-亚硝基哌啶(NPIP)、*N*-亚硝基吡咯烷(NPYR)、*N*-亚硝基吗啉(NMOR)列为2B类致癌物。

肉制品中的*N*-亚硝胺主要与肉制品的生产原料、加工方式等因素有关。当肉制品处于酸性环境中时,亚硝酸盐会和蛋白质分解产生的仲胺进行亚硝化形成*N*-亚硝胺^[12]^。传统风干牦牛肉是藏民族极具特色的生食肉制品,以新鲜牦牛肉为原料,经分割、切条后在低温干燥条件下自然风干而成^[13]^。除了传统的加工制作工艺外,目前还有蒸煮或者烤制加工的熟制风干牦牛肉。然而由于制作工艺的不规范以及自然环境条件的影响,风干牦牛肉易产生致癌物*N*-亚硝胺,因此加强风干牦牛肉中*N*-亚硝胺的监管尤为重要。

对于食品尤其是肉制品中亚硝胺测定的样品前处理方法主要包括水蒸气蒸馏^[14]^、分散液液微萃取^[15,16]^和固相萃取^[17,18]^。这些方法需要的样品量大,操作过程复杂且耗时。QuEChERS方法已广泛应用于检测领域^[19,20]^。Qiu等^[21]^采用QuEChERS样品前处理方法和气相色谱-串联质谱法(GC-MS/MS)测定了咸鱼中的9种挥发性*N*-亚硝胺,平均回收率为86.9%~113.1%。一步式QuEChERS样品制备管分外管和内管,涡旋振荡与离心同时进行,以实现手动QuEChERS方法中样品提取和净化的功能,该前处理技术可简化程序进而提高样品制备的效率,提高试验的重复性^[22]^。谢瑜杰等^[23]^采用新型一步式QuEChERS设备用于检测牛肉中的25种磺胺类药物残留,方法简单快捷。目前已有基于GC-MS/MS对肉制品中*N*-亚硝胺进行分析的报道,但针对风干牦牛肉中亚硝胺的检测鲜有报道。

本研究以民族特色食品风干牦牛肉中的15种*N*-亚硝胺类化合物为研究对象,利用一步式QuEChERS技术对样品进行提取及净化,采用GC-MS/MS对*N*-亚硝胺类化合物进行分析测定,相对于其他肉制品中*N*-亚硝胺的测定方法,本方法操作简单,人为影响因素小,可极大地提高实验效率,同时为风干牦牛肉及其他肉制品中*N*-亚硝胺的测定和法规完善提供方法支持和数据借鉴。

## 1 实验部分

### 1.1 仪器、试剂与材料

Agilent 8890A-7000E气相色谱-三重四极杆质谱仪(美国Agilent公司); QuEChERS自动样品制备系统Sio-8512、自动QuEChERS整合管(包括外管和净化内管)(北京本立科技公司); Milli-Q超纯水机(美国Millipore公司); TRIOTM-1N涡旋混合器(日本ASONE公司); Auto EVA 80高通量全自动平行浓缩仪(厦门睿科仪器有限公司)。

15种*N*-亚硝胺标准溶液:NDMA (1002.4 μg/mL)、NMEA (1002.0 μg/mL)、NDEA (999.8 μg/mL)、*N*-亚硝基乙基异丙基胺(NEIPA, 1006.0 μg/mL)、*N*-亚硝基二异丙胺(NDIPA, 998.0 μg/mL)、*N*-亚硝基二丙胺(NDPA, 1001.6 μg/mL) *N*-亚硝基二异丁胺(NDIBA, 1000.9 μg/mL)、NDBA (1000.1 μg/mL)、*N*-亚硝基甲基苯胺(NMPhA, 1000.0 μg/mL)、*N*-亚硝基乙基苯胺(NEPhA, 997.9 μg/mL)、NPIP(1001.8 μg/mL)、NPYR (999.5 μg/mL)、NMOR (1000.7 μg/mL)、*N*-亚硝基二苯胺(NDPhA, 1004.8 μg/mL)、*N*-亚硝基二苄胺(NDBzA, 1004.0 μg/mL) (天津阿尔塔科技有限公司);乙腈、乙酸乙酯、二氯甲烷(色谱纯,美国Fisher公司);无水硫酸镁、氯化钠(分析纯,国药集团化学试剂有限公司); 十八烷基硅烷(C_18_)、*N*-丙基乙二胺(PSA)(上海安谱实验科技股份有限公司)。

10份代表性风干牦牛肉样品均购自电商平台。将肉干切成肉丁,取适量放入粉碎机均质粉碎,并于-18 ℃冷冻备用。

### 1.2 标准溶液的配制

分别移取一定量的15种*N*-亚硝胺标准溶液于10 mL容量瓶中,乙腈定容至刻度,得到质量浓度为10.0 mg/L混合标准溶液,于-18 ℃避光保存;根据需要,移取适量10.0 mg/L混合标准溶液,用乙腈稀释,配制所需浓度的标准工作液,于4 ℃避光保存。向空白基质样品中添加适量15种*N*-亚硝胺类化合物混合标准溶液,配制成0.1、0.5、1、2、5、10、20、50、100和200 μg/L的系列空白基质匹配校准溶液,现用现配。

### 1.3 样品前处理

称取粉碎后的样品5 g(精确至0.01 g)于离心管外管中,加入10 mL去离子水、4颗锆珠,涡旋1 min,再加入15 mL乙腈、4 g无水硫酸镁、1.0 g氯化钠,将净化内管(含500 mg无水硫酸镁、25 mg C_18_和50 mg PSA)与外管拧紧,放入全自动QuEChERS前处理设备,以4000 r/min转速振荡、离心各5 min,温度4 ℃,重复2次。取内管中1.5 mL上清液于30 ℃下氮吹至0.5 mL以下,用乙腈定容至0.5 mL,取200 μL,经0.2 μm滤膜过滤,用于GC-MS/MS测定。

### 1.4 分析条件

#### 1.4.1 色谱条件

色谱柱:Agilent DB-HeavyWAX气相色谱柱(30 m×0.25 mm×0.25 μm);载气:氦气(99.999%);载气流速为1.0 mL/min;柱温:初始温度40 ℃,保持0.5 min,以15 ℃/min升温至190 ℃,再以40 ℃/min升温至260 ℃,保持5 min;进样口温度:220 ℃;进样体积:1 μL;进样方式:不分流进样。

#### 1.4.2 质谱条件

溶剂延迟:5.5 min;离子化方式:电子轰击(EI);电离能量70 eV;离子源温度:250 ℃;四极杆温度:150 ℃;进样口温度:250 ℃;碰撞气:高纯氮气(99.999%);检测模式:多反应监测(MRM)。15种*N*-亚硝胺的保留时间和质谱参数见表1。

**表1:** 15种*N*-亚硝胺的保留时间和质谱参数

No.	Compound	CAS No.	Retention time/min	Ion pairs (m/z)	CEs/eV		
1	N-nitrosodimethylamine (NDMA, N-亚硝基二甲胺)	62-75-9	6.46	74.0>44.1^*^, 74.0>42.1	3,		19
2	N-nitrosomethylethylamine (NMEA, N-亚硝基甲乙胺)	10595-95-6	6.88	88.0>71.1^*^, 88.0>42.1	2,		18
3	N-nitrosodiethylamine (NDEA, N-亚硝基二乙胺)	55-18-5	7.16	102.1>85.0^*^, 102.1>44.0	2,		11
4	N-nitrosoethylisopropylamine (NEIPA, N-亚硝基乙基异丙基胺)	16339-04-1	7.47	116.0>99.0^*^, 116.0>44.0	5,		15
5	N-isopropyl-N-nitroso-2-propanamine (NDIPA, N-亚硝基二异丙胺)	601-77-4	7.70	130.1>42.1^*^, 130.1>88.0	10,		5
6	N-nitrosodipropylamine (NDPA, N-亚硝基二丙胺)	621-64-7	8.36	130.1>113.0^*^, 130.1>43.0	1,		9
7	N-nitrosodiisobutylamine (NDIBA, N-亚硝基二异丁胺)	997-95-5	8.56	115.0>84.0^*^, 115.0>57.0	3,		5
8	N-nitrosodibutylamine (NDBA, N-亚硝基二正丁胺)	924-16-3	9.85	116.0>99.0^*^, 116.0>74.0	2,		8
9	N-methyl-N-phenylnitrous amide (NMPhA, N-亚硝基甲基苯胺)	614-00-6	9.92	106.0>77.0^*^, 106.0>51.0	20,		40
10	N-ethyl-N-nitrosoaniline (NEPhA, N-亚硝基乙基苯胺)	612-64-6	9.94	121.0>106.0^*^, 121.0>77.0	10,		35
11	N-nitrosopiperidine (NPIP, N-亚硝基哌啶)	100-75-4	10.10	114.1>84.0^*^, 114.1>41.0	5,		13
12	N-nitrosopyrrolidine (NPYR, N-亚硝基吡咯烷)	930-55-2	10.33	100.0>55.0^*^, 100.0>43.0	5,		9
13	N-nitrosomorpholine (NMOR, N-亚硝基吗啉)	59-89-2	10.75	116.0>86.0^*^, 116.0>56.0	1,		11
14	N-nitrosodiphenylamine (NDPhA, N-亚硝基二苯胺)	86-30-6	14.30	169.1>113.1^*^, 169.1>99.1	17,		30
15	N-nitrosodibenzylamine (NDBzA, N-亚硝基二苄胺)	5336-53-8	17.03	226.1>181.0^*^, 226.1>103.0	5,		20

* Quantitative ion; CE: collision energy.

## 2 结果与讨论

### 2.1 气相色谱条件的优化

分别考察了3种不同极性的气相色谱柱(HP-5MS UI (30 m×0.25 mm×0.25 μm)、DB-1701 (30 m×0.25 mm×0.25 μm)和DB-HeavyWAX (30 m×0.25 mm×0.25 μm))对15种*N*-亚硝胺类化合物的分离效果。结果表明,采用弱极性的HP-5MS UI色谱柱时,*N*-亚硝胺类化合物的保留效果较差,特别是NDMA和NEMA的保留时间较短,均在3 min内出峰,容易产生与溶剂或基质等分离不完全的现象。NDEA和NEIPA在HP-5MS UI色谱柱中保留不佳,且存在一定程度的峰拖尾现象(图1a),不适合准确定量。当采用DB-1701色谱柱时,10种*N*-亚硝胺的响应值低于DB-HeavyWAX,例如,DB-HeavyWAX中NDEA和NEIPA的响应值约为DB-1701色谱柱的3倍(图1b、1c)。采用强极性DB-HeavyWAX色谱柱时,能够很好地分离15种*N*-亚硝胺类化合物,且峰形良好(图2)。因此,本实验选择DB-HeavyWAX色谱柱对15种*N*-亚硝胺进行检测。

**图1:**
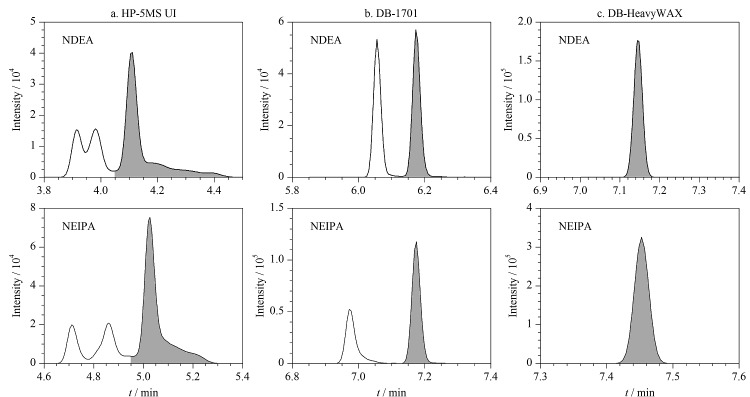
HP-5MS UI、DB-1701和DB-HeavyWAX色谱柱上NDEA和NEIPA(100 μg/L)的色谱图

**图2:**
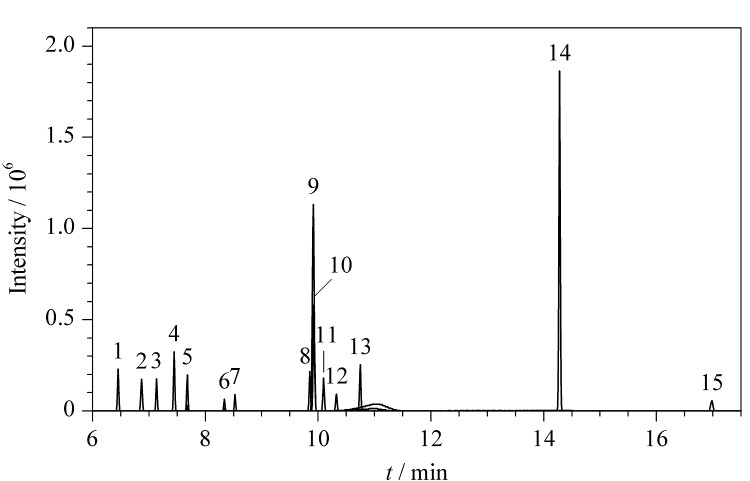
15种*N*-亚硝胺标准品的色谱图(100 μg/L)

### 2.2 样品前处理条件优化

#### 2.2.1 水化体积的优化

风干牦牛肉晾晒时间长,水分含量低,打碎后为干料,加水进行水化会使干燥肉干中的孔隙相对膨胀进而促进化学提取^[24]^。当水化体积为6 mL时,样品才能完全浸润,所以选取加水量为6、8、10和12 mL对水化体积进行优化。如图3所示,在6~10 mL内,随着水化体积增加,15种*N*-亚硝胺的回收率显著增加,10 mL时15种*N*-亚硝胺的回收率最佳,因此,本实验选择水化体积为10 mL。

**图3:**
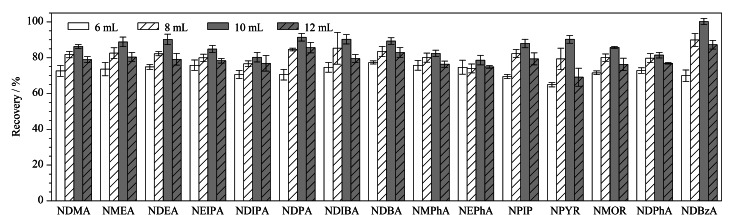
水化体积对15种*N*-亚硝胺回收率的影响(*n*=3)

#### 2.2.2 萃取溶剂的优化

萃取溶剂是影响目标化合物回收率的关键因素,因此结合基质的性质考察了乙酸乙酯、二氯甲烷和乙腈^[25⇓⇓⇓-29]^对15种*N*-亚硝胺提取效果的差异。结果表明,乙酸乙酯易使极性小的脂肪、油脂等共萃取,从而对仪器造成污染;二氯甲烷提取效果差且毒性强。如图4所示,由于油脂在乙腈中的溶解度较低,乙腈比其他萃取溶剂产生了更好的回收率。因此,最终选择乙腈作为萃取溶剂用于后续实验。

**图4:**
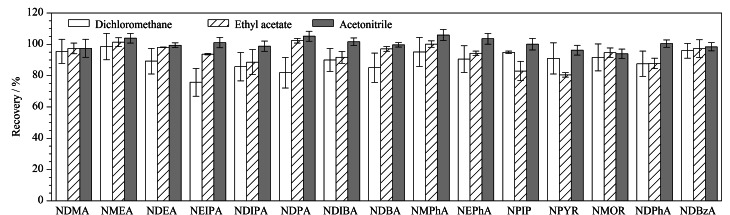
萃取溶剂对15种*N*-亚硝胺回收率的影响(*n*=3)

#### 2.2.3 盐的优化

通过加入适量或不同组合的盐可调控有机相中的水分含量,提高目标化合物的回收率。*N*-亚硝胺提取常使用无水MgSO_4_和NaCl的混合盐^[24,25,30]^,因此,首先考察了不同用量的NaCl(0.5、1.0、1.5和2.0 g)对15种*N*-亚硝胺回收率的影响。如图5a所示,随着NaCl用量从0.5 g增加到2.0 g,几乎所有*N*-亚硝胺的回收率均呈现先增加后下降的趋势,因此NaCl用量为1.0 g时*N*-亚硝胺回收率最佳。无水MgSO_4_能够结合大量的水,促使化合物向有机相转移和分配,在1.0 g NaCl的基础上考察不同用量的无水MgSO_4_(3.0、4.0、5.0和6.0 g)对实验结果的影响。如图5b所示,不同用量的MgSO_4_均具有良好的萃取效果,其中4.0 g MgSO_4_和1.0 g NaCl混合盐效果优于其他3种加盐量,因此,本实验选择4.0 g MgSO_4_和1.0 g NaCl为最终添加量。

**图5:**
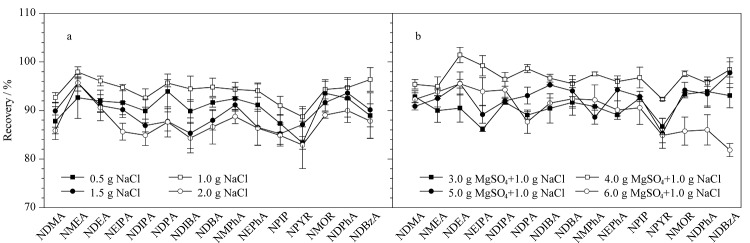
(a)NaCl和(b)MgSO_4_用量对15种*N*-亚硝胺回收率的影响(*n*=3)

#### 2.2.4 净化填料的优化

风干牦牛肉基质成分复杂,含盐量高,并含有多种氨基酸等风味成分,共提取组分可能会导致基质增强或基质抑制,且污染检测仪器,从而影响目标分析物的准确定量。根据风干牦牛肉的特性,本研究选择QuEChERS方法最常用的无水MgSO_4_、C_18_和PSA用于提取净化风干牦牛肉中的15种*N*-亚硝胺。

如图6所示,C_18_和PSA的组合相较于单独使用无水MgSO_4_、C_18_和PSA均表现出更好的回收率。因此,选择C_18_+PSA用于净化步骤。使用无水MgSO_4_去除水分,同时使用C_18_和PSA混合吸附剂来有效去除提取物中的干扰。对于C_18_+PSA的用量,首先比较C_18_不同用量对回收率的影响,如图7a所示,25 mg C_18_情况下15种*N*-亚硝胺的回收率最佳。固定C_18_用量后比较不同PSA用量对回收率的影响,如图7b所示,50 mg PSA时,回收率最佳。综合各目标物的提取效率和实验成本,最终选择500 mg无水MgSO_4_+25 mg C_18_+50 mg PSA作为净化填料。

**图6:**
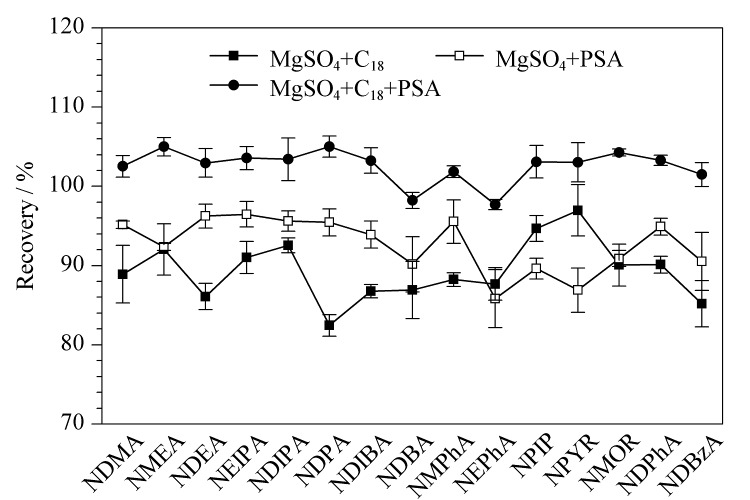
净化填料对15种*N*-亚硝胺回收率的影响(*n*=3)

**图7:**
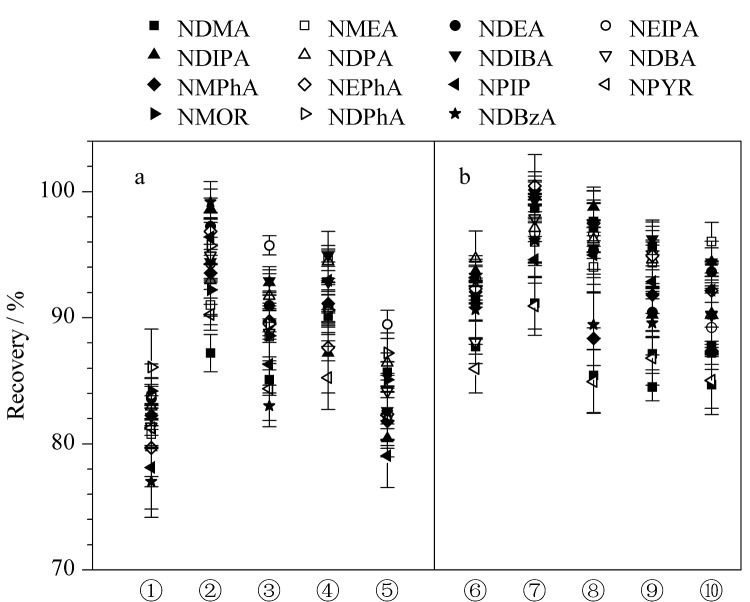
不同(a)C_18_、(b)PSA用量对15种*N*-亚硝胺回收率的影响(*n*=3)

#### 2.2.5 氮吹程度的优化

当净化后的上清液氮吹至完全干燥时,如图8所示,15种*N*-亚硝胺的回收率较低(1.4%~70.8%)。

**图8:**
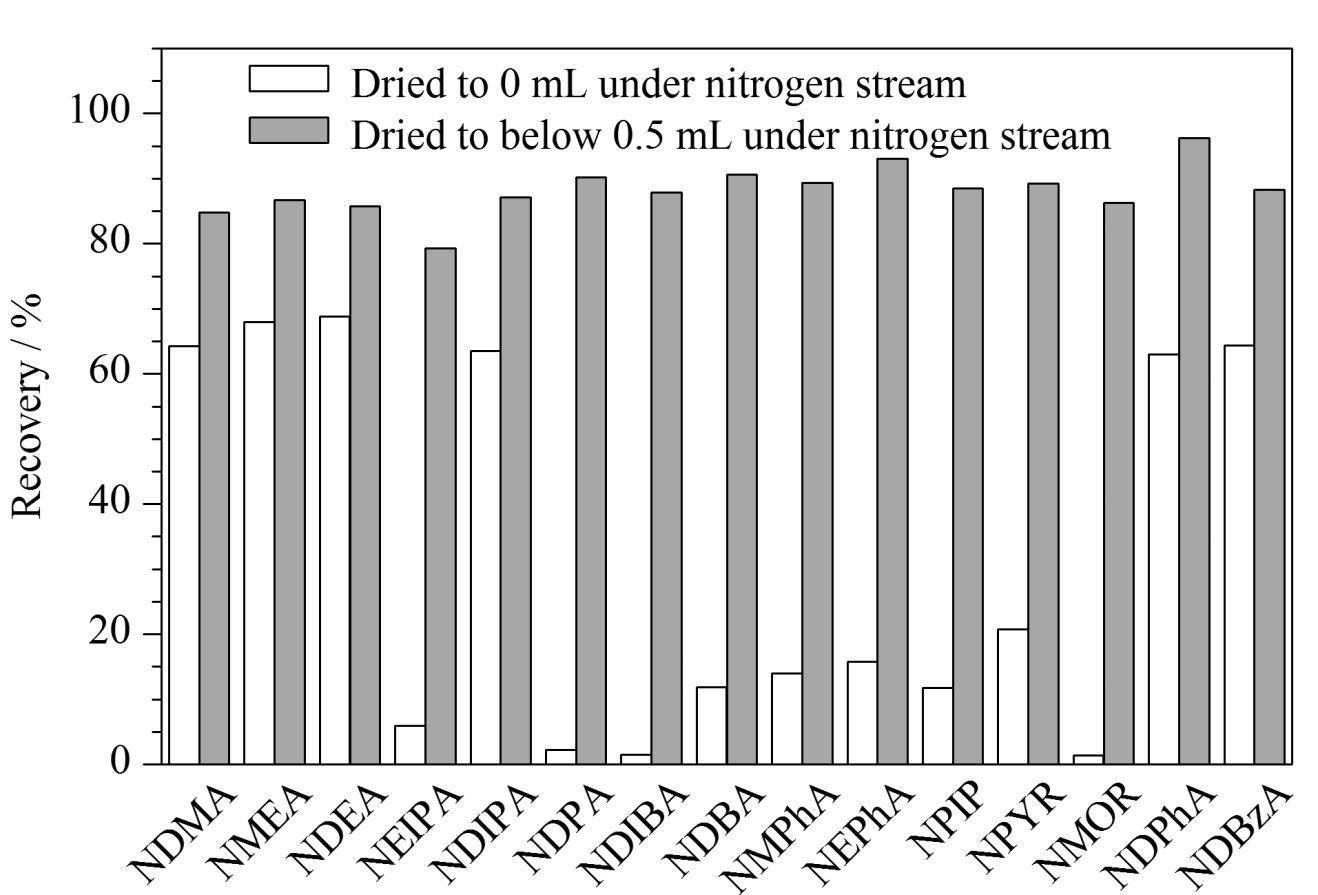
氮吹程度对15种*N*-亚硝胺回收率的影响(*n*=3)

据相关文献报道,当提取液不完全干燥时,*N*-亚硝胺可获得良好的回收率^[10,31]^。因此,选择将上清液在温和的氮气流下氮吹至0.5 mL以下,然后重新用乙腈定容至0.5 mL,取200 μL,用于GC-MS/MS分析,进而提高方法的准确度。结果表明,在这种氮气干燥模式下,15种*N*-亚硝胺的回收率为79.3%~96.2%。

### 2.3 基质效应

基质效应是指样品基质中目标分析物以外的成分对检测结果的影响,基质效应的计算公式如下^[32]^:ME=(基质匹配标准曲线斜率/溶剂标准曲线斜率-1)×100%,当|ME|≤20%时表现为弱基质效应,当20%<|ME|≤50%时表现为中等基质效应,当|ME|>50%时表现为强基质效应^[33]^。如图9所示,15种*N*-亚硝胺在生制风干牦牛肉中均表现为弱基质效应,表明本实验方法灵敏度高,但在熟制风干牦牛肉中3种*N*-亚硝胺表现为中等基质效应,因此,本研究采用基质匹配曲线以补偿基质效应对目标化合物的干扰,进一步提高检测方法的准确性。

**图9:**
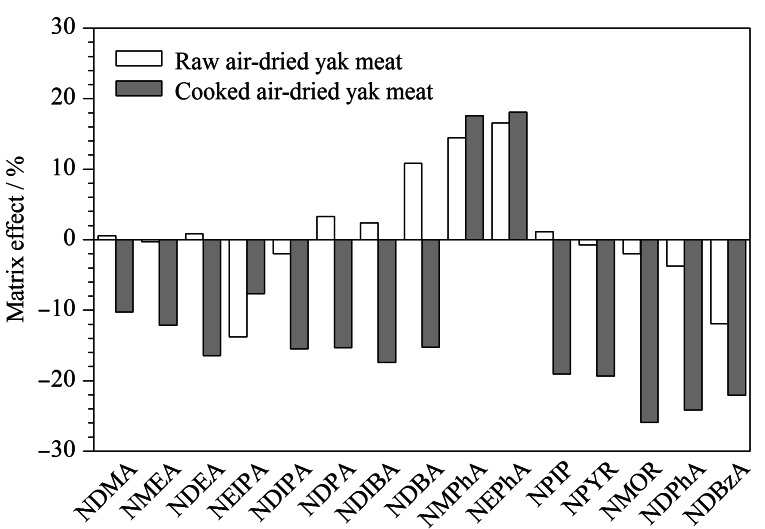
15种*N*-亚硝胺的基质效应

### 2.4 方法学验证

#### 2.4.1 线性范围、相关系数、检出限和定量限

在最优条件下对方法学参数进行验证,采用基质匹配标准曲线法定量检测,以峰面积为纵坐标,相应质量浓度为横坐标得出各化合物的线性方程。如表2所示,15种*N*-亚硝胺的相关系数(*r*^2^)为0.9990~0.9998,表明目标物在相应质量浓度范围内线性关系良好。以信噪比(*S/N*)≥3对应的添加水平作为检出限(LOD),以*S/N*≥10对应的添加水平作为定量限(LOQ),15种*N*-亚硝胺的LOD和LOQ范围分别为0.05~0.20 μg/kg和0.10~0.50 μg/kg。其LOD和LOQ水平均低于孔祥一等^[34]^报道的QuEChERS法结合同位素定量测定动物源性食品中9种*N*-亚硝胺类化合物的LOD(0.03~0.50 μg/kg)和LOQ(0.10~1.00 μg/kg),表明该方法的灵敏度较高。线性范围大于赵庄等^[29]^报道的QuEChERS法测定酸肉样品中10种挥发性*N*-亚硝胺类化合物的线性范围(1~100 μg/L)。

**表2:** 15种*N*-亚硝胺的线性范围、相关系数、检出限、定量限、平均回收率和相对标准偏差(*n*=6)

Compound	Linear range/ (μg/L)	r^2^	LOD/ (μg/kg)	LOQ/ (μg/kg)	Average recoveries (RSDs)/%													
					1LOQ				2LOQ						10LOQ				
					Raw air- dried yak meat		Cooked air- dried yak meat			Raw air- dried yak meat			Cooked air- dried yak meat		Raw air- dried yak meat		Cooked air- dried yak meat		
NDMA	0.1-200	0.9992	0.05	0.10	91.3 (4.7)		91.7 (10.5)				89.8 (3.9)		84.6 (16.0)			83.2 (0.9)		97.7 (2.8)	
NMEA	0.1-200	0.9997	0.05	0.10	95.3 (5.3)		82.2 (7.6)				93.4 (2.5)		89.0 (11.6)			85.0 (2.0)		85.8 (3.8)	
NDEA	0.1-200	0.9995	0.05	0.10	94.1 (6.5)		88.6 (10.3)				95.2 (4.4)		84.6 (6.8)			84.0 (4.9)		100.0 (3.8)	
NEIPA	0.2-200	0.9997	0.10	0.20	79.4 (2.9)		82.3 (3.7)				106.3 (4.9)		95.2 (5.8)			85.8 (2.8)		86.2 (2.1)	
NDIPA	0.5-200	0.9998	0.20	0.50	93.5 (7.1)		98.8 (4.5)				94.0 (1.8)		95.9 (5.6)			87.7 (1.2)		91.9 (7.6)	
NDPA	0.1-200	0.9997	0.05	0.10	100.4 (6.5)		93.1 (9.1)				90.9 (7.8)		80.6 (11.4)			87.7 (6.6)		83.0 (4.5)	
NDIBA	0.1-200	0.9997	0.05	0.10	102.1 (10.4)		82.6 (4.9)				106.7 (9.6)		98.2 (14.1)			89.2 (1.6)		106.7 (8.4)	
NDBA	0.2-200	0.9994	0.10	0.20	86.5 (4.3)		87.0 (4.2)				92.1 (1.0)		86.5 (8.9)			92.0 (0.8)		91.4 (9.5)	
NMPhA	0.2-200	0.9997	0.10	0.20	95.3 (4.5)		93.3 (7.1)				109.5 (3.4)		97.6 (5.9)			94.4 (7.8)		101.6 (6.5)	
NEPhA	0.5-200	0.9995	0.20	0.50	98.4 (2.7)		93.8 (6.0)				103.7 (3.8)		91.7 (7.2)			102.8 (7.6)		106.9 (3.6)	
NPIP	0.5-200	0.9998	0.20	0.50	90.7 (8.1)		80.4 (5.7)				102.0 (3.1)		92.1 (8.5)			89.1 (1.8)		91.9 (7.0)	
NPYR	0.5-200	0.9991	0.20	0.50	95.8 (6.0)		92.7 (6.3)				85.6 (4.2)		89.1 (8.8)			96.8 (5.5)		93.5 (12.0)	
NMOR	0.2-200	0.9990	0.10	0.20	97.8 (1.4)		102.1 (4.7)				94.1 (1.2)		82.7 (9.7)			90.1 (3.3)		83.6 (9.7)	
NDPhA	0.5-200	0.9996	0.20	0.50	101.0 (2.3)		101.8 (5.3)				104.2 (3.6)		106.2 (6.9)			96.9 (2.3)		110.6 (3.2)	
NDBzA	0.5-200	0.9995	0.20	0.50	94.6 (10.2)		99.1 (12.8)				102.4 (3.4)		86.5 (4.0)			99.2 (3.8)		102.1 (6.2)	

#### 2.4.2 回收率和精密度

为评价方法的准确度与精密度,取空白基质,按1倍LOQ、2倍LOQ和10倍LOQ 3个添加水平进行回收试验,每个水平重复6次,计算各添加水平的平均回收率和RSD。如表2所示,生风干牦牛肉中3个添加水平下的平均回收率分别为79.4%~102.1%(1倍LOQ)、85.6%~109.5%(2倍LOQ)、83.2%~102.8%(10倍LOQ); RSD为0.8%~10.4%。熟风干牦牛肉中3个添加水平下的平均回收率分别为80.4%~102.1%(1倍LOQ)、80.6%~106.2%(2倍LOQ)、83.0%~110.6%(10倍LOQ); RSD为2.1%~16.0%。通过1天内测定3个加标水平下的6个平行样品得到日内精密度,连续3天测定3个加标水平下的6个平行样品得到日间精密度。以RSD表示的日内、日间精密度分别不大于8.1%和18.2%。表明所建立方法具有良好的准确度和精密度,可同时满足两种加工方式的风干牦牛肉中*N*-亚硝胺的检测要求。

### 2.5 实际样品分析

应用本研究建立的方法,对10份风干牦牛肉样品进行分析,其中5份样品为生制风干牦牛肉,另5份样品为熟制风干牦牛肉。结果如表3所示,两种风干牦牛肉中NDMA的含量均未超过GB 2762-2022《食品安全国家标准 食品中污染物限量》规定值(肉制品(肉类罐头除外)3.0 μg/kg,熟肉干制品3.0 μg/kg)。此外生制风干牦牛肉中*N*-亚硝胺检出率和平均含量分别为40%~60%和0.08~0.79 μg/kg,熟制风干牦牛肉中*N*-亚硝胺检出率和平均含量分别为40%~100%和0.23~20.18 μg/kg;熟制风干牦牛肉中*N*-亚硝胺的检出率和平均含量均高于传统生制风干牦牛肉,这可能与熟制风干牦牛肉的复杂加工工艺有关^[35]^。

**表3:** 10份风干牦牛肉样品中15种*N*-亚硝胺的含量水平

Compound	Detection rates/%			Contents/(μg/kg) (average (min-max))	
	Raw air- dried yak meat	Cooked air- dried yak meat		Raw air- dried yak meat	Cooked air- dried yak meat
NDMA	60	100		0.79 (ND-1.76)	0.99 (0.90-1.14)
NMEA	ND	ND		ND	ND
NDEA	ND	ND		ND	ND
NEIPA	ND	ND		ND	ND
NDIPA	ND	ND		ND	ND
NDPA	ND	ND		ND	ND
NDIBA	40	60		0.45 (ND-1.67)	0.65 (ND-2.24)
NDBA	40	100		0.08 (ND-0.22)	0.23 (0.15-0.30)
NMPhA	40	100		0.17 (ND-0.49)	10.54 (0.36-20.10)
NEPhA	ND	80		ND	20.18 (11.08-39.20)
NPIP	ND	ND		ND	ND
NPYR	ND	40		ND	2.40 (2.34-9.66)
NMOR	ND	ND		ND	ND
NDPhA	40	100		0.41 (ND-1.24)	1.01 (0.78-1.18)
NDBzA	ND	ND		ND	ND

ND: not detected.

## 3 结论

本研究应用一步式QuEChERS法结合GC-MS/MS建立了一种风干牦牛肉中15种*N*-亚硝胺的分析方法,并应用于实际样品的检测。该方法操作简便,自动化程度高,灵敏可靠,适用于风干牦牛肉中15种*N*-亚硝胺的测定。该方法的建立有助于为风干牦牛肉中*N*-亚硝胺控制技术的研发提供方法支持,同时可为其他肉制品中*N*-亚硝胺的测定提供参考。
